# Polypharmacy in outpatients with relapsing-remitting multiple sclerosis: A single-center study

**DOI:** 10.1371/journal.pone.0211120

**Published:** 2019-01-24

**Authors:** Niklas Frahm, Michael Hecker, Uwe Klaus Zettl

**Affiliations:** Neuroimmunology Section, Department of Neurology, University of Rostock, Rostock, Germany; Charite Universitatsmedizin Berlin, GERMANY

## Abstract

**Background:**

Multiple sclerosis (MS) is an immune-mediated disease of the central nervous system. Given the chronic and heterogenous nature of the disease, treatment with various therapies is a frequent scenario in clinical practice. In persons with chronic morbidity such as MS patients, polypharmacy can give rise to considerable health problems.

**Objectives:**

The aim of the present study was to examine the frequency of polypharmacy among relapsing-remitting (RR) MS patients as well as to analyse sociodemographic and clinical factors, which might be associated with polypharmacy (use of five or more medications). Differences in medication between MS patients with and without secondary illnesses (PwSI and Pw/oSI), between men and women and between patients with and without polypharmacy (PwP and Pw/oP) were examined.

**Methods:**

For 145 RRMS outpatients, we prospectively collected data by means of anamnesis, patient records, clinical examination and a structured patient interview. This was followed by comparative analyses of various patient subgroups (PwP vs. Pw/oP, PwSI vs. Pw/oSI, men vs. women).

**Results:**

The proportion of included MS patients with polypharmacy (use of ≥5 medications) was 30.3%. PwP were significantly older than Pw/oP (45.9 vs. 41.7 years), had a lower level of education and showed a significantly higher median EDSS score (3.0 vs. 2.0). Comorbidities (*p*<0.001; odds ratio [OR] = 6.293) and higher EDSS scores (*p* = 0.029; OR = 1.440) were associated with a higher risk of polypharmacy. The proportion of polypharmacy among PwSI was approximately four times higher than among Pw/oSI (46.8% vs. 11.8%). Particularly in the use of antihypertensives, gastrointestinal drugs and dietary supplements, there were differences between Pw/oP and PwP.

**Conclusion:**

We found a high burden of polypharmacy in patients with RRMS. This particularly applies to more severely disabled MS patients who suffer from comorbidities.

## Introduction

Multiple sclerosis (MS) is an immune-mediated disease of the central nervous system, which causes pathological demyelination, axonal damage and loss of synapses [1]. It can lead to multiple persisting symptoms. MS can occur in any age group, although most diagnoses are made between the ages of 20 and 49 years [2]. More than 2.3 million people are affected globally, underlining the socioeconomic burden of this disease [3,4]. Investigations have shown that genetic and environmental factors play a role in the manifestation of MS [5,6].

The introduction of interferon-beta-1b preparations as the first of the disease-modifying drugs (DMDs) in 1993 marked a significant step forward in the development of and research into new immunomodulatory drugs for MS treatment [7–10]. Along with DMDs, symptomatic therapies contribute to improve the patients’ individual quality of life [11]. In the course of disease, various symptoms such as fatigue [12], coordination disturbances [13], emotional disturbances [14], pain [15], sensory disturbances [16], paresis and spasticity [17] can manifest.

In view of this complexity of treatment scenarios, the factor of polypharmacy needs to be taken into account. The most common definition of polypharmacy is the use of five or more medications [18–23]. For years and even decades, an increasing frequency of polypharmacy has been observed among patients in the general population. For instance, in the Tayside region of Scotland, the number of patients with polypharmacy doubled from 1995 to 2010 [24]. Older persons are often particularly affected, as they are more likely to suffer from comorbidities, leading to an increase in the number of medications taken. Ignoring the factor of polypharmacy in drug therapy can give rise to rehospitalizations [25], serious drug interactions [24], lack of adherence due to medication complexity [26], cognitive decline, rising socioeconomic costs and side effects [27]. So far, there are only few studies on partial aspects of polypharmacy in MS [28–31].

The aim of the present study was to examine polypharmacy in outpatients with relapsing-remitting MS (RRMS) and to evaluate possible associations with sociodemographic and clinical-neurological factors. Additionally, the occurrence of polypharmacy was examined in relation to comorbidities while including the whole range of medications taken.

## Materials and methods

This cross-sectional study was conducted at the Department of Neurology at the University of Rostock. The data were gathered between March 2017 and April 2018 using three sources of information: anamnesis and patient records, clinical examination and a structured patient interview. These three sources are described in the following as “examination”. Inclusion criteria for the study were a confirmed diagnosis of relapsing-remitting MS according to the revised McDonald criteria from 2010 [32] and admission to the outpatient department. With verbal informed consent, 147 patients attended the examination, two of whom declined to participate due to personal reasons. Thus, 145 patients were included in the study. This study was approved by the University of Rostock’s ethics committee (permit number A 2014–0089) and carried out in line with the Declaration of Helsinki. Verbal consent was documented by the interviewer. The consent procedure was approved by the ethics committee who considered that since the study was non-invasive and used only interviewing of participants, verbal consent would be adequate.

### Data acquisition

The patients were prospectively studied regarding sociodemographic, clinical-neurological and pharmacological aspects. The full dataset is given in the supplementary material (S1 Table).

The sociodemographic data included age, gender, number of years in school (not including years in higher education or training), educational level, employment status, partnership and place of residence (rural community–less than 5000 residents, provincial town– 5000 till 20000 residents, medium-sized town– 20000 till 100000 residents, city–more than 100000 residents) as well as number of children and siblings.

The clinical-neurological data were operationalized by Kurtzke’s Expanded Disability Status Scale (EDSS), which categorizes the degree of disability of MS patients [33] and by disease duration since the time of the initial diagnosis. Moreover, data on the presence of comorbidities were gathered.

To obtain the pharmacological data, the patients’ medication plans were consulted. These contained the trade name of each medication, the respective indication, active ingredients, potency, dosage and route of administration.

To ensure the completeness of the collected data, a structured patient interview and a review of the medical records were conducted for each patient. Only medications which were actually taken by the patient were regarded in the analysis.

### Medication analysis

To enable a more precise analysis, the medications were evaluated according to three criteria.

The first criterion entailed a distinction between long-term and as-needed (*pro re nata*; PRN) medications. Long-term medications are taken daily or at regular intervals, for instance once a week or once a month, to treat illnesses or complaints. PRN medications are taken at irregular intervals to treat acute or sporadic complaints.

The second criterion referred to the prescription status of the respective medication, with a distinction being made between prescription-only and over-the-counter (OTC) medications.

The third criterion was based on the therapeutic objective. A distinction was made between the categories DMDs, specific treatment of MS symptoms and treatment of a secondary illness or further complaints. The class of DMDs comprises the immunomodulatory active substances approved for the long-term treatment of MS [34,35]. Symptomatic drugs aim at a targeted alleviation of particular symptoms of MS, such as spasticity or pain. The category of treatment of a secondary illness or further complaints includes all medications that are not taken for the treatment of MS.

### Definition of polypharmacy and comorbidities

As the use of five or more medications represents the most commonly reported definition of polypharmacy [18–23], this was used as the threshold in order to compare patients without polypharmacy (Pw/oP) to those with polypharmacy (PwP). Accordingly, patients who took zero to four medications were counted as Pw/oP and those who took five or more medications were counted as PwP. Additionally, we examined whether differences emerged if the categorization into Pw/oP and PwP was made according to the total number of medications used (i.e. the sum of both long-term and PRN medications) or according to the number of long-term medications only.

A comorbidity was registered if it was listed in the patient records. If the records were outdated, its mention in the patient interview and the targeted pharmacological treatment of this comorbidity were used for the assessment. Patients with no comorbidities were categorized as patients without secondary illnesses (Pw/oSI). By contrast, patients with secondary illnesses (PwSI) had at least one comorbidity.

### Statistical analysis

PASW Statistics 18 (IBM) was used for all statistical analyses. All patient data were anonymized prior to entry into the database. The data were tested for normal distribution. To compare the different patient groups (PwP, Pw/oP, PwSI, Pw/oSI, men and women), we used two-sample two-tailed Student’s t-tests, Mann-Whitney U tests, Chi-square tests and Fisher’s exact tests for the descriptive univariable analyses. Subsequently, a multivariable binary logistic regression with stepwise forward variable selection based on the likelihood ratio was conducted to determine the subset of associations between polypharmacy (defined according to the total number of medications taken) as response variable and eight sociodemographic variables (age, sex, years of school, educational level, partnership, place of residence, number of children and siblings) and three clinical-neurological variables (EDSS, disease duration and comorbidities) as explanatory variables with the statistically most significant predictive value. This analysis considers more complex data dependencies and yields odds ratios (OR) for those variables that are sequentially entered into the model. The significance level was set at α = 0.05. False discovery rate (FDR) correction was applied to take into account alpha error inflation in the case of multiple testing [36].

## Results

### Study population

The patients’ sociodemographic data are shown in Table 1. The patient cohort comprised a total of 145 patients with a confirmed diagnosis of RRMS. Considering the patients’ gender, 73.8% (N = 107) were women and 26.2% (N = 38) were men. The average age was 43.0 years (range 19 to 68 years). Women were significantly younger than men (*p* = 0.003). More than half of the 145 patients (51.7%) were still in employment at the time of the study, while 39.3% were already retired. Most patients were in a partnership and the majority had at least one child and at least one sibling.

**Table 1:** Sociodemographic data of the examined MS patients.

	Total population		Female		Male		*p*-value
N	145		107		38		
	N (%)		N (%)		N (%)		
**Age (Years)**	19–68^R^	43.0 (11.4)^a^	19–67^R^	41.3 (11.4)^a^	22–68^R^	47.7 (9.8)^a^	**0.003**^t^
≤ 29	19 (13.1)		18 (16.8)		1 (2.6)		
30–39	44 (30.3)		35 (32.7)		9 (23.7)		
40–49	35 (24.1)		23 (21.5)		12 (31.6)		
50–59	38 (26.2)		25 (23.4)		13 (34.2)		
≥ 60	9 (6.2)		6 (5.6)		3 (7.9)		
**School years**	8–16^R^	10.0^b^	8–16^R^	10.0^b^	8–13^R^	10.0^b^	0.198^U^
**Educational level**							0.326^Chi^
No training	3 (2.1)		3 (2.8)		0 (0.0)		
Skilled worker	91 (62.8)		68 (63.6)		23 (60.5)		
Technical college	8 (5.5)		4 (3.7)		4 (10.5)		
University	43 (29.7)		32 (29.9)		11 (28.9)		
**Employ-ment status**							0.304^Chi^
In training	4 (2.8)		4 (3.7)		0 (0.0)		
Employed	75 (51.7)		51 (47.7)		24 (63.2)		
Unemployed	4 (2.8)		3 (2.8)		1 (2.6)		
Retiree	57 (39.3)		44 (41.1)		13 (34.3)		
Others	5 (3.4)		5 (4.7)		0 (0.0)		
**Partnership**							0.522^Fi^
Yes	108 (74.5)		78 (72.9)		30 (78.9)		
No	37 (25.5)		29 (27.1)		8 (21.1)		
**Place of residence**							**0.033**^Chi^
Rural community	39 (26.9)		34 (31.8)		5 (13.2)		
Provincial town	26 (17.9)		19 (17.8)		7 (18.4)		
Medium-sized town	15 (10.3)		13 (12.1)		2 (5.3)		
City	65 (44.8)		41 (38.3)		24 (63.2)		
**Number of children**	0–3^R^	1^b^	0–3^R^	1^b^	0–3^R^	1^b^	0.384^U^
0	42 (29.0)		31 (29.0)		11 (28.9)		
1	49 (33.8)		33 (30.8)		16 (42.1)		
2	45 (31.0)		35 (32.7)		10 (26.3)		
3	9 (6.2)		8 (7.5)		1 (2.6)		
**Number of siblings**	0–13^R^	1^b^	0–13^R^	1^b^	0–5^R^	1^b^	0.080^U^
0	15 (10.3)		12 (11.2)		3 (7.9)		
1	83 (57.2)		65 (60.7)		18 (47.4)		
≥ 2	47 (32.4)		30 (28.0)		17 (44.7)		

MS, multiple sclerosis; N, number of patients.

^a^ mean value (standard deviation);

^b^ median;

^Chi^ Chi-square test;

^Fi^ Fisher’s exact test;

^R^ range;

^t^ two-sample two-tailed Student’s t-test;

^U^ Mann-Whitney U test.

The clinical-neurological data are summarized in Table 2. The EDSS scores ranged from 1.0 to 7.5, with a median of 2.5. The median disease duration was 11 years and varied between 1 and 36 years, although a substantial proportion of the patients (30.3%) had a disease duration between one and five years. PwSI (53.1%) were slightly predominant over Pw/oSI (46.9%). Comparing the EDSS scores, disease duration and the presence of comorbidity between men and women, there were no significant differences.

**Table 2:** Clinical data of the examined MS patients.

	Total population		Female		Male		*p*-value
N	145		107		38		
	N (%)		N (%)		N (%)		
**EDSS**	1.0–7.5^R^	2.5^b^	1.0–7.5^R^	2.5^b^	1.0–6.0^R^	3.0^b^	0.059^U^
1.0	6 (4.1)		5 (4.7)		1 (2.6)		
1.5	29 (20.0)		23 (21.5)		6 (15.8)		
2.0	29 (20.0)		24 (22.4)		5 (13.2)		
2.5	16 (11.0)		11 (10.3)		5 (13.2)		
3.0	26 (17.9)		19 (17.8)		7 (18.4)		
3.5	14 (9.7)		11 (10.3)		3 (7.9)		
4.0	15 (10.3)		8 (7.5)		7 (18.4)		
>4.0	10 (6.9)		6 (5.5)		4 (10.5)		
**Disease duration (Years)**	1–36^R^	11.4 (7.1)^a^	1–36^R^	10.8 (7.2)^a^	3–29^R^	13.2 (6.7)^a^	0.072^t^
1–5	44 (30.3)		38 (35.5)		6 (15.8)		
6–10	25 (17.2)		17 (15.9)		8 (21.1)		
11–15	34 (23.4)		23 (21.5)		11 (28.9)		
16–20	28 (19.3)		20 (18.7)		8 (21.1)		
≥ 21	14 (9.7)		9 (8.4)		5 (13.2)		
**Comorbi-dities**							0.259^Fi^
Pw/oSI	68 (46.9)		47 (43.9)		21 (55.3)		
PwSI	77 (53.1)		60 (56.1)		17 (44.7)		

EDSS, expanded disability status scale; MS, multiple sclerosis; N, number of patients; PwSI, patients with secondary illnesses; Pw/oSI, patients without secondary illnesses.

^a^ mean value (standard deviation);

^b^ median;

^Fi^ Fisher’s exact test;

^R^ range;

^t^ two-sample two-tailed Student’s t-test;

^U^ Mann-Whitney U test.

### Analysis of factors related to polypharmacy

**Fig 1:**
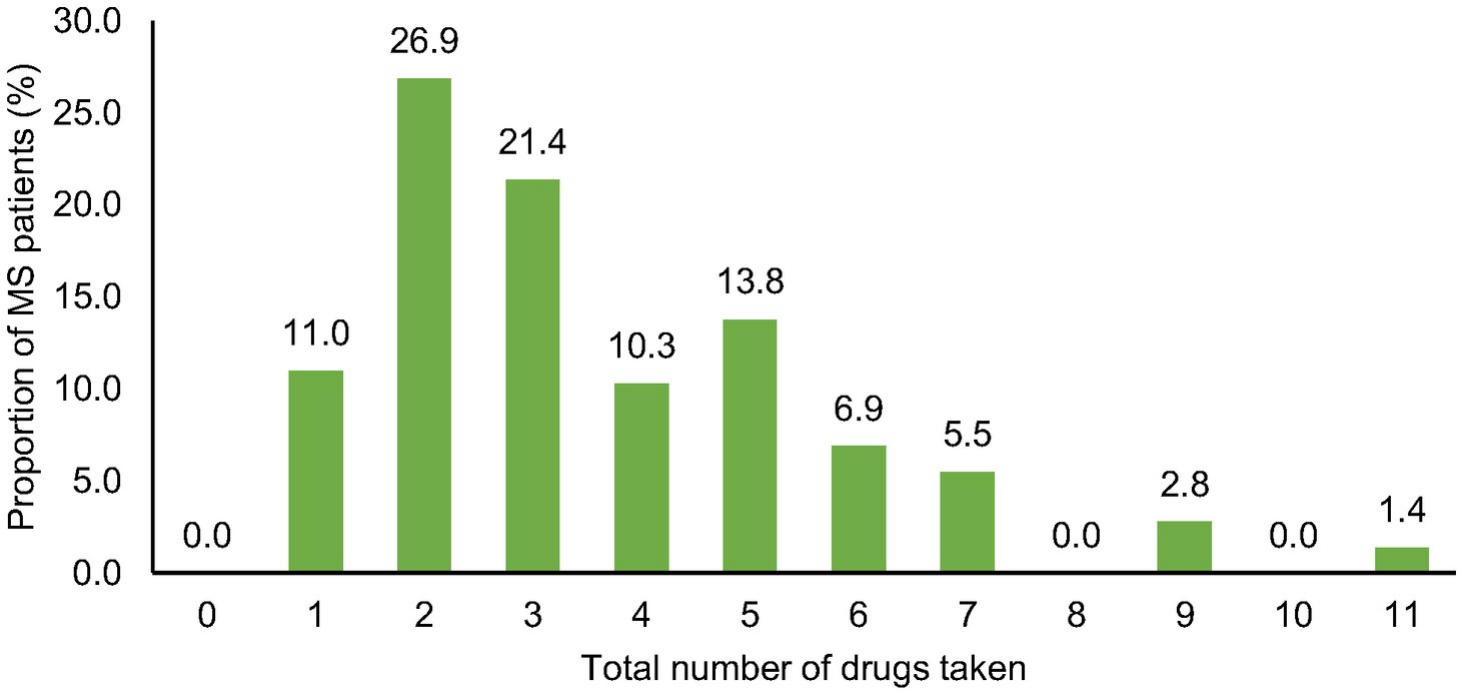
Distribution of the total number of medications taken by the examined MS patients. Only few MS patients (16.6%, N = 24) in this study took more than five drugs. Most frequently, MS patients took two medications (26.9%, N = 39). MS, multiple sclerosis.

When analysing the patient cohort according to the total number of medications taken (long-term and PRN drugs), 30.3% (N = 44) were categorized as PwP. Considering only the number of long-term medications, 20.7% (N = 30) were categorized as PwP. The patients took on average 3.6 (SD 2.1) medications overall, with a range from 1 to 11 (Fig 1). Thus, every participant took at least one medication, although this was not an inclusion criterion for the present study.

When polypharmacy was categorized based on all medications, PwP were significantly older than Pw/oP (*p* = 0.042) (Table 3). Moreover, there were significantly more retirees in the PwP group, while a greater number of Pw/oP were employed (*p* = 0.034). After FDR correction of the *p*-values, no significant differences remained for the comparisons of the sociodemographic data between Pw/oP and PwP.

**Table 3:** Comparison of sociodemographic data between patients with and without polypharmacy.

	Polypharmacy (all medications)		Polypharmacy (long-term medications only)	
	Pw/oP vs. PwP	*p*-value	Pw/oP vs. PwP	*p*-value
**N**	101 vs. 44		115 vs. 30	
**Age (Years)**^a^	41.7 (11.1) vs. 45.9 (11.5)	**0.042**^t^	41.2 (11.0) vs. 49.7 (10.3)	**<0.001**^t^
**School years**^a^	11.1 (1.4) vs. 10.7 (1.2)	0.118^t^	11.1 (1.4) vs. 10.5 (1.0)	**0.008**^t^
**Educational level**^c^		0.169^Chi^		0.673^Chi^
No training	3 (3.0) vs. 0 (0.0)		3 (2.6) vs. 0 (0.0)	
Skilled worker	58 (57.4) vs. 33 (75.0)		70 (60.9) vs. 21 (70.0)	
Technical college	7 (6.9) vs. 1 (2.3)		7 (6.1) vs. 1 (3.3)	
University	33 (32.7) vs. 10 (22.7)		35 (30.4) vs. 8 (26.7)	
**Employment status**^c^		**0.034**^Chi^		**0.039**^Chi^
In training	3 (3.0) vs. 1 (2.3)		4 (3.5) vs. 0 (0.0)	
Employed	60 (59.4) vs. 15 (34.1)		65 (56.5) vs. 10 (33.3)	
Unemployed	3 (3.0) vs. 1 (2.3)		4 (3.5) vs. 0 (0.0)	
Retiree	31 (30.7) vs. 26 (59.1)		38 (33.0) vs. 19 (63.3)	
Other	4 (4.0) vs. 1 (2.3)		4 (3.5) vs. 1 (3.3)	
**Partnership**^c^		0.836^Fi^		0.248^Fi^
Yes	76 (75.2) vs. 32 (72.7)		83 (72.2) vs. 25 (83.3)	
No	25 (24.8) vs. 12 (27.3)		32 (27.8) vs. 5 (16.7)	
**Gender**^c^		1.000^Fi^		0.643^Fi^
Female	74 (73.3) vs. 33 (75.0)		86 (74.8) vs. 21 (70.0)	
Male	27 (26.7) vs. 11 (25.0)		29 (25.2) vs. 9 (30.0)	
**Place of residence**^c^		0.721^Chi^		0.202^Chi^
Rural community	29 (28.7) vs. 10 (22.7)		31 (27.0) vs. 8 (26.7)	
Provincial town	17 (16.8) vs. 9 (20.5)		20 (17.4) vs. 6 (20.0)	
Medium-sized town	9 (8.9) vs. 6 (13.6)		9 (7.8) vs. 6 (20.0)	
City	46 (45.5) vs. 19 (43.2)		55 (47.8) vs. 10 (33.3)	
**Number of children**^b^	1 vs. 1	0.589^U^	1 vs. 1	0.462^U^
**Number of siblings**^b^	1 vs. 1	0.587^U^	1 vs. 1	0.311^U^

N, number of patients; PwP, patients with polypharmacy; Pw/oP, patients without polypharmacy.

^a^ mean value (standard deviation);

^b^ median;

^c^ number of patients (%);

^Chi^ Chi-square test;

^Fi^ Fisher’s exact test;

^t^ two-sample two-tailed Student’s t-test;

^U^ Mann-Whitney U test.

Analysing polypharmacy without including PRN drugs, there were similar results in age (*p*<0.001) and employment status (*p* = 0.039). Additionallyt, Pw/oP also had significantly more years in school than PwP. Apart from the significant age difference, no further differences remained significant following FDR correction.

**Fig 2:**
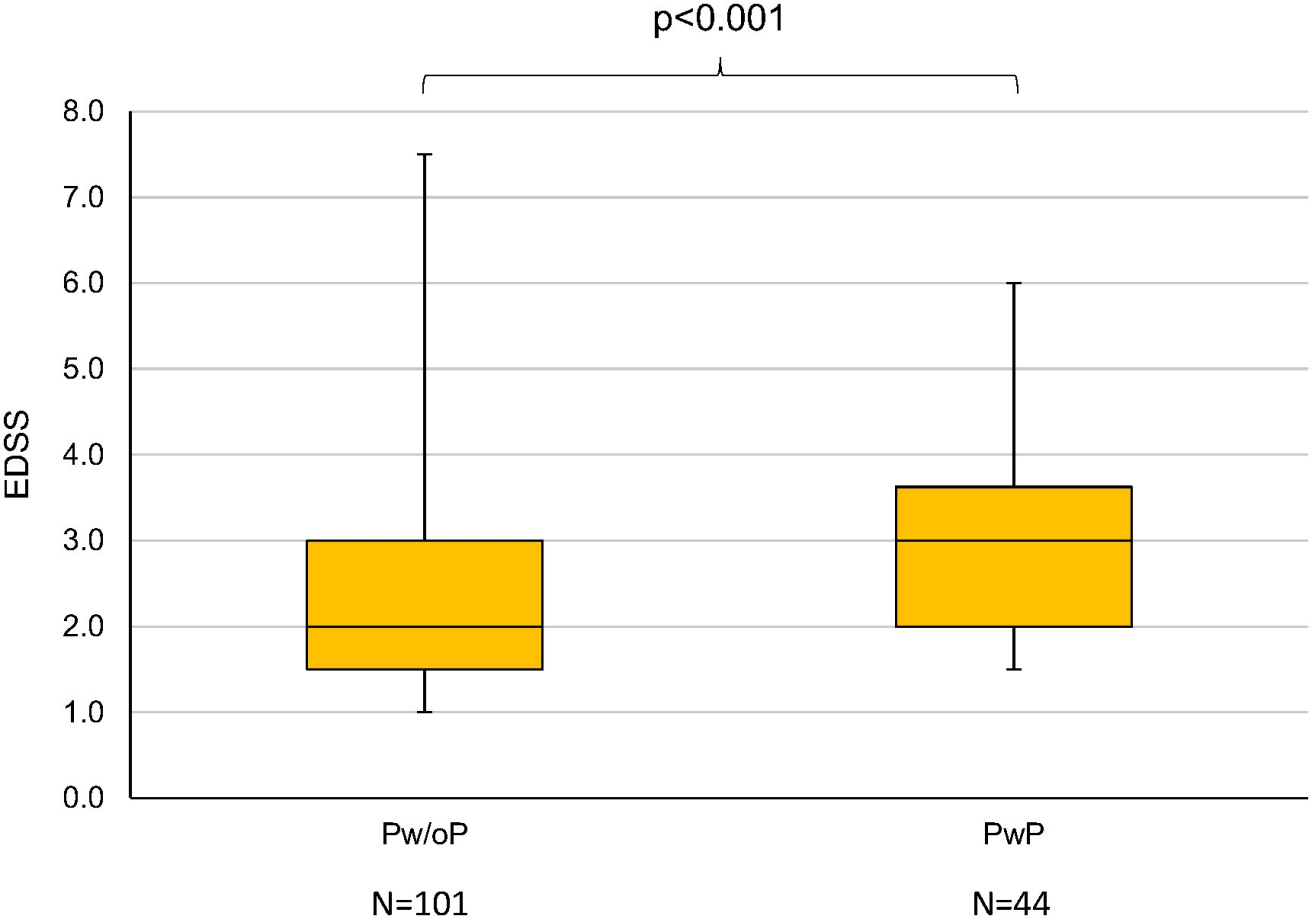
Comparison of EDSS scores between Pw/oP and PwP. Boxplot of the degree of disability of the patients stratified by the presence of polypharmacy according to the total number of medications taken (Pw/oP: N = 101; PwP: N = 44). The boxes mark the upper and lower quartiles of the EDSS scores per group. The medians are indicated by horizontal lines. The whiskers extend to the minimum and maximum values. PwP had, on average, a significantly higher level of disability than Pw/oP, as evaluated by EDSS (Mann-Whitney U test: *p*<0.001). EDSS, expanded disability status scale; N, number of patients; *p*, *p*-value; PwP, patients with polypharmacy; Pw/oP, patients without polypharmacy.

Pw/oP had lower EDSS median scores than PwP (Fig 2), both when considering all medications (*p*<0.001) and when considering long-term medications only (*p* = 0.040) (Table 4). There was no significant difference with regard to disease duration. Moreover, it was apparent that the proportion of MS patients with comorbidities was twice as high in PwP as in Pw/oP. According to Fisher’s exact test, the comparison of the number of patients with and without comorbidities between Pw/oP and PwP yielded *p*-values ≤ 0.001.

**Table 4:** Comparison of clinical data between patients with and without polypharmacy.

	Polypharmacy (total medications)		Polypharmacy (long-term medications only)	
	Pw/oP vs. PwP	*p*-value	Pw/oP vs. PwP	*p*-value
**N**	101 vs. 44		115 vs. 30	
**EDSS**^b^	2.0 vs. 3.0	**<0.001**^U^	2.5 vs. 3.0	**0.040**^U^
**Disease duration (Years)**^b^	11.0 vs. 11.0	0.784^U^	10.0 vs. 13.0	0.248^U^
**Comorbidities**^c^		**<0.001**^Fi^		**<0.001**^Fi^
Pw/oSI	60 (59.4) vs. 8 (18.2)		67 (58.3) vs. 1 (3.3)	
PwSI	41 (40.6) vs. 36 (81.8)		48 (41.7) vs. 29 (96.7)	

EDSS, expanded disability status scale; N, number of patients; PwP, patients with polypharmacy; PwSI, patients wih secondary illnesses; Pw/oP, patients without polypharmacy; Pw/oSI, patients without secondary illnesses.

^b^ median;

^c^ number of patients (%);

^Fi^ Fisher’s exact test;

^U^ Mann-Whitney U test.

**Fig 3:**
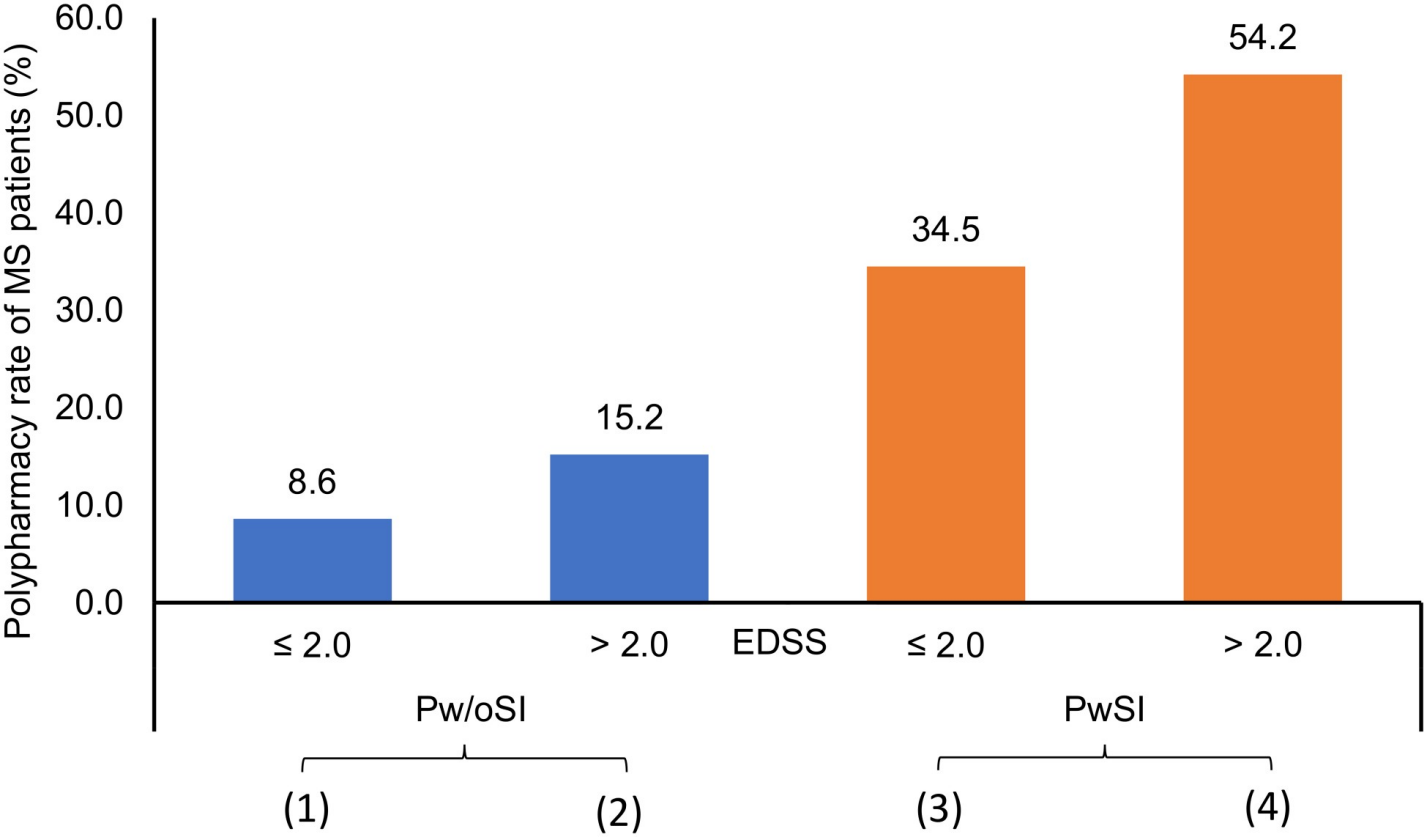
Comparison of polypharmacy rates between patients with and without comorbidities at different levels of disability. The patients were split into four groups according to EDSS score and the presence of comorbidities. (1) Pw/oSI with EDSS ≤ 2.0 (that is, below the median of the total population): Three of the 35 patients had polypharmacy (8.6%). (2) Pw/oSI with EDSS > 2.0: Five of the 33 patients had polypharmacy (15.2%). (3) PwSI with EDSS ≤ 2.0: Ten of the 29 patients had polypharmacy (34.5%). (4) PwSI with EDSS > 2.0: Twenty-six of the 48 patients had polypharmacy (54.2%). Considering all four polypharmacy rates, the highest proportion of polypharmacy occurred in MS patients with comorbidity and high EDSS scores. Using both factors in a logistic regression model of polypharmacy yielded an overall prediction accuracy rate of 71.7%. EDSS, expanded disability status scale; MS, multiple sclerosis; Pw/oSI, patients without secondary illnesses; PwSI, patients with secondary illnesses.

A multivariable logistic regression was calculated to obtain a minimal predictive model of the risk of polypharmacy. The model was built in two steps. Thus, 2 out of the 11 explanatory variables were included by the stepwise forward selection procedure: Higher EDSS scores and the presence of comorbidities were significantly associated with a higher risk of polypharmacy (EDSS score with *p* = 0.029 and OR = 1.440; comorbidities with *p*<0.001 and OR = 6.293) (Fig 3). The prediction accuracy of the fitted model for correctly distinguishing PwP was 71.7%. All other variables, i.e. age, school years, educational level, partnership status, gender, place of residents, number of children, number of siblings and disease duration, were not included in the model further.

**Fig 4:**
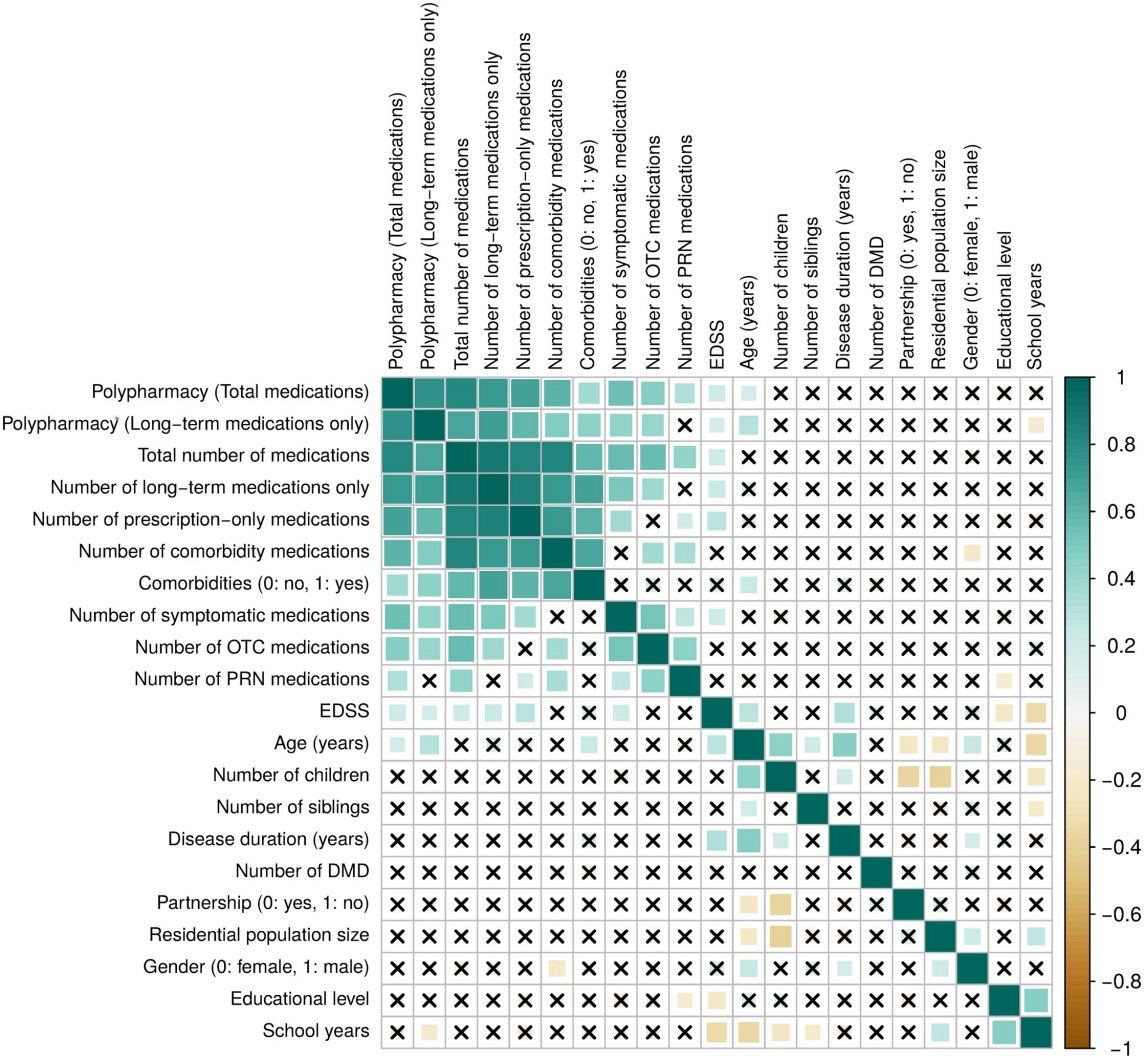
Correlation plot for the associations between variables and polypharmacy status. The symmetric correlation matrix was created using the “corrplot” R package. The colours represent the degree of pairwise correlation regarding Spearman’s rank correlation coefficient (rho). The crosses indicate absence of correlation (asymptomatic t approximation *p*-values > 0.05). For example, EDSS and the number of symptomatic drugs as well as age and comorbidities correlated with each other. DMD, disease-modifying drug; EDSS, expanded disability status scale; OTC, over-the-counter; PRN, *pro re nata*.

An analysis of Spearman’s rank correlation coefficients revealed significant pairwise interdependencies between several variables as shown in the correlation matrix in Fig 4. For instance, the presence of comorbidities was significantly positively correlated with age.

### Medication analysis

The medications used were subdivided into 24 medication groups in order to have an overview about the frequencies of use of different medication groups and to examine potential differences between PwP and Pw/oP. The most frequent medication group in our cohort of RRMS patients was the group of DMDs. Then dietary supplements, analgesics, osteoporosis drugs and contraceptives followed (Table 5).

**Table 5:** Frequency of medication use in MS patients.

		Polypharmacy (total medications)				Polypharmacy (long-term medications only)			
	All^a^	Pw/oP^a^	PwP^a^	*p*^Fi^	FDR^Fi^	Pw/oP^a^	PwP^a^	*p*^Fi^	FDR^Fi^
**N**	145	101	44			115	30		
**DMDs**	95.9	96.0	95.5	1.000	1.000	95.7	96.7	1.000	1.000
**Dietary supplements**	33.8	24.8	54.5	**0.001**	**0.006**	27.0	60.0	**0.001**	**0.010**
**Analgesics**	29.0	19.8	50.0	**0.001**	**0.006**	27.0	36.7	0.366	0.482
**Osteoporosis drugs**	22.8	17.8	34.1	0.051	0.106	18.3	40.0	**0.016**	**0.046**
Contraceptives*	22.1	20.8	25.0	0.664	0.802	24.3	13.3	0.227	0.313
**Thyroid drugs**	15.9	11.9	25.0	0.081	0.142	12.2	30.0	**0.025**	0.062
**Antidepressants**	12.4	8.9	20.5	0.061	0.118	8.7	26.7	**0.013**	**0.042**
**Antihypertensives**	11.7	5.0	27.3	**<0.001**	**<0.001**	5.2	36.7	**<0.001**	**<0.001**
**Gastrointestinal drugs**	11.7	5.0	27.3	**<0.001**	**<0.001**	7.8	26.7	**0.009**	**0.033**
**Aconuresis drugs**	9.0	5.0	18.2	**0.022**	0.053	5.2	23.3	**0.006**	**0.029**
**Anticonvulsants**	7.6	3.0	18.2	**0.003**	**0.012**	3.5	23.3	**0.002**	**0.012**
**Thrombosis prophylactics**	7.6	3.0	18.2	**0.003**	**0.012**	3.5	23.3	**0.002**	**0.012**
**Muscle relaxants**	6.9	2.0	18.2	**0.001**	**0.006**	5.2	13.3	0.216	0.313
**Antiallergics**	5.5	5.0	6.8	0.699	0.811	6.1	3.3	1.000	1.000
**Sedatives**	4.8	3.0	9.1	0.200	0.302	3.5	10.0	0.155	0.281
**Antiinfectives**	4.1	1.0	11.4	**0.010**	**0.026**	0.9	16.7	**0.001**	**0.010**
**Eye drops**	2.8	0.0	9.1	**0.008**	**0.026**	0.9	10.0	**0.028**	0.062
**Statins**	2.8	1.0	6.8	0.083	0.142	0.9	10.0	**0.028**	0.062
**Asthma drugs**	2.1	0.0	6.8	**0.027**	0.060	0.0	10.0	**0.008**	**0.033**
**Insulin**	2.1	1.0	4.5	0.219	0.302	2.6	0.0	1.000	1.000
**Parkinson’s disease drugs**	2.1	1.0	4.5	0.219	0.302	0.9	6.7	0.109	0.211
**Psoriasis drugs**	1.4	2.0	0.0	1.000	1.000	1.7	0.0	1.000	1.000
**Arthritis IT**	1.4	0.0	4.5	0.091	0.147	0.0	6.7	**0.042**	0.087
**Others**:	6.9	3.0	15.9	**0.009**	**0.026**	5.2	13.3	0.216	0.313
Fampridine	4.1	1.0	11.4	**0.010**	**0.026**	3.5	6.7	0.604	0.762
Clozapine	0.7	1.0	0.0	1.000	1.000	0.9	0.0	1.000	1.000
Ginkgo leaves dry extract	0.7	0.0	2.3	0.303	0.382	0.0	3.3	0.207	0.313
Modafinil	0.7	0.0	2.3	0.303	0.382	0.0	3.3	0.207	0.313
Rizatriptan	0.7	1.0	0.0	1.000	1.000	0.9	0.0	1.000	1.000

DMDs, disease-modifying drugs; FDR, false discovery rate; IT, immunotherapy; Pw/oP, patients without polypharmacy; PwP, patients with polypharmacy.

^a^ frequency of use of medication groups (%);

^Fi^ Fisher’s exact test;

* including hormone replacement therapy.

Considering polypharmacy according to overall medications, the most important differences between Pw/oP and PwP were in dietary supplements, analgesics, muscle relaxants, antihypertensives and gastrointestinal drugs (FDR≤0.006). Considering long-term medications only, significant differences occurred e.g., for in gastrointestinal drugs, antihypertensives, antidepressants, osteoporosis drugs and dietary supplements (FDR<0.05). For antiallergics, contraceptives, DMDs, insulin, Parkinson’s disease drugs, psoriasis drugs and sedatives, there were no significant differences for the stratification of patients by polypharmacy (FDR>0.05).

The analysis of the pharmacological data revealed significant differences for almost all medication categories with FDR<0.05 (Table 6). In the group of PwP, the number of medications taken was, on average, two to three times as high as in the group of Pw/oP. Counted with repetitions, the sum of all medications taken by the 145 MS patients amounted to 521. Although the group of Pw/oP in our study was clearly larger than the group of PwP (101 vs. 44), the majority of medications (52.8%) were taken by PwP. When evaluating polypharmacy with including PRN drugs, the only category for which no significant differences emerged between Pw/oP and PwP was DMDs (*p* = 0.871). Analysing polypharmacy without including PRN drugs, only DMDs (*p* = 0.804) and PRN medications (*p* = 0.597) did not show significant differences between Pw/oP and PwP.

**Table 6:** Comparison of pharmacological data between patients with and without polypharmacy.

	Polypharmacy (total medications)		Polypharmacy (long-term medications only)	
	Pw/oP vs. PwP	*p*-value	Pw/oP vs. PwP	*p*-value
**N**	101 vs. 44		115 vs. 30	
**Total medications**^a^	2.4 (0.9) vs. 6.2 (1.6)	**<0.001**^U^	2.8 (1.3) vs. 6.6 (1.8)	**<0.001**^U^
**Long-term medications**^a^	2.0 (0.9) vs. 4.0 (1.8)	**<0.001**^U^	2.2 (1.0) vs. 5.8 (1.3)	**<0.001**^U^
**PRN medications**^a^	0.4 (0.6) vs. 1.2 (1.2)	**<0.001**^U^	0.6 (0.8) vs. 0.8 (1.0)	0.597^U^
**Prescription-only medications**^a^	1.8 (0.9) vs. 4.3 (1.5)	**<0.001**^U^	2.1 (1.2) vs. 4.6 (1.5)	**<0.001**^U^
**OTC medications**^a^	0.6 (0.7) vs. 1.9 (1.6)	**<0.001**^U^	0.7 (0.9) vs. 2.0 (1.6)	**<0.001**^U^
**DMD**^a^	1.0 (0.2) vs. 1.0 (0.3)	0.871^U^	1.0 (0.2) vs. 1.0 (0.2)	0.804^U^
**Symptomatic medications**^a^	0.4 (0.6) vs. 1.8 (1.4)	**<0.001**^U^	0.5 (0.8) vs. 1.9 (1.5)	**<0.001**^U^
**Comorbidity medications**^a^	1.1 (0.9) vs. 3.5 (2.0)	**<0.001**^U^	1.3 (1.2) vs. 3.7 (2.1)	**<0.001**^U^

DMD, disease-modifying drug; N, number of patients; OTC, over-the-counter; PRN, *pro re nata*; PwP, patients with polypharmacy; Pw/oP, patients without polypharmacy.

^a^ mean value (standard deviation) of the number of drugs taken per patient;

^U^ Mann-Whitney U test.

With regard to the routes of drug administration, PwP used more frequently peroral (*p* = 0.006 and *p* = 0.041) and conjunctival drugs (*p* = 0.008 and *p* = 0.028) than the Pw/oP group (Table 7). These differences remained significant after correcting the *p*-values for multiple testing (FDR< 0.05).

**Table 7:** Comparison of routes of drug administration between patients with and without polypharmacy.

	Polypharmacy (total medications)			Polypharmacy (long-term medications only)		
Route of administration	Pw/oP^a^	PwP^a^	*p*-value^Fi^	Pw/oP^a^	PwP^a^	*p*-value^Fi^
**N**	101	44		115	30	
**buccal**	0.0	4.5	0.091	0.9	3.3	0.372
**conjunctival**	0.0	9.1	**0.008**	0.9	10.0	**0.028**
**cutaneous**	1.0	2.3	0.516	1.7	0.0	1.000
**intramuscular**	5.9	6.8	1.000	6.1	6.7	1.000
**intravenous**	38.6	34.1	0.709	39.1	30.0	0.241
**nasal**	1.0	0.0	1.000	0.9	0.0	1.000
**peroral**	85.1	100.0	**0.006**	87.0	100.0	**0.041**
**pulmonary**	1.0	6.8	0.083	0.9	10.0	**0.028**
**rectal**	0.0	2.3	0.303	0.0	3.3	0.207
**subcutaneous**	36.6	36.4	1.000	36.5	36.7	0.574
**sublingual**	2.0	2.3	1.000	1.7	3.3	0.504
**vaginal**	0.0	4.5	0.091	0.9	3.3	0.372

N, number of patients; PwP, patients with polypharmacy; Pw/oP, patients without polypharmacy.

^a^ frequency of routes of drug administration (%);

^Fi^ Fisher’s exact test.

### Comparison of MS patients with and without comorbidities

We next examined differences with respect to the presence of comorbidities (Table 8). Seventy-seven patients suffered from MS and one or more secondary illnesses (53.1%). Table 9 shows the distribution of comorbidities in the whole MS cohort (N = 145). The most frequent comorbidities were autoimmune diseases with thyroid diseases being on top. Pw/oSI were, on average, significantly younger than PwSI (*p* = 0.005). This difference remained significant after correcting the *p*-values according to FDR. Otherwise no other sociodemographic and clinical-neurological variables were significantly associated with comorbidity. The rate of polypharmacy in the PwSI group (46.8%) was much higher than in the Pw/oSI group (11.8%).

**Table 8:** Comparison of sociodemographic data between patients with and without comorbidities.

	Pw/oSI		PwSI		*p*-value
N	68		77		
	N (%)		N (%)		
**Age (Years)**	19–61^R^	40.2 (11.0)^a^	22–68^R^	45.5 (11.2)^a^	**0.005**^t^
≤ 29	14 (20.6)		5 (6.5)		
30–39	21 (30.9)		23 (29.9)		
40–49	17 (25.0)		18 (23.4)		
50–59	15 (22.1)		23 (29.9)		
≥ 60	1 (1.5)		8 (10.4)		
**Gender**					0.259^Fi^
Female	47 (69.1)		60 (77.9)		
Male	21 (30.9)		17 (22.1)		
**School years**	8–13^R^	11.0 (1.4)^a^	9–16^R^	10.9 (1.3)^a^	0.476^t^
**Educational level**					0.276^Chi^
No training	3 (4.4)		0 (0.0)		
Skilled worker	43 (63.2)		48 (62.3)		
Technical college	4 (5.9)		4 (5.2)		
University	18 (26.5)		25 (32.5)		
**Employment status**					0.701^Chi^
In training	3 (4.4)		1 (1.3)		
Employed	36 (52.9)		39 (50.6)		
Unemployed	2 (2.9)		2 (2.6)		
Retiree	24 (35.3)		33 (42.9)		
Other	3 (4.4)		2 (2.6)		
**Partnership**					0.344^Fi^
Yes	48 (70.6)		60 (77.9)		
No	20 (29.4)		17 (22.1)		
**Place of residence**					0.473^Chi^
Rural community	17 (25.0)		22 (28.6)		
Provincial town	15 (22.1)		11 (14.3)		
Medium-sized town	5 (7.4)		10 (13.0)		
City	31 (45.6)		34 (44.2)		
**Number of children**	0–3^R^	1^b^	0–3^R^	1^b^	0.904^U^
0	24 (35.3)		18 (23.4)		
1	15 (22.1)		34 (44.2)		
2	25 (36.8)		20 (26.0)		
3	4 (5.9)		5 (6.5)		
**Number of siblings**	0–8^R^	1^b^	0–13^R^	1^b^	0.296^U^
0	4 (5.9)		11 (14.3)		
1	40 (58.8)		43 (55.8)		
≥ 2	24 (35.3)		23 (29.9)		

MS, multiple sclerosis; N, number of patients; PwP, patients with polypharmacy; PwSI, patients with secondary illnesses; Pw/oP, patients without polypharmacy; Pw/oSI, patients without secondary illnesses; R, range.

^a^ mean value (standard deviation);

^b^ median;

^Chi^ Chi-square test;

^Fi^ Fisher’s exact test;

^R^ range;

^t^ two-sample two-tailed Student’s t-test;

^U^ Mann-Whitney U test.

**Table 9:** Comorbidities of the MS cohort (N = 145).

Comorbidities	N (%)
**Autoimmune diseases**	32 (22.2)
Thyroid	23 (15.9)
Type 1 diabetes mellitus	3 (2.1)
Bronchial asthma	3 (2.1)
Arthritis	2 (1.4)
Psoriasis	1 (0.7)
**Neurologic diseases**	20 (13.8)
Sleep disturbance	6 (4.1)
Migraine	5 (3.4)
Other headache	3 (2.1)
Epilepsy	2 (1.4)
Restless legs syndrome	2 (1.4)
Raynaud’s syndrome	1 (0.7)
Forgetfulness	1 (0.7)
**Cardiovascular diseases**	19 (13.1)
Hypertonia	17 (11.7)
Coronary heart disease	1 (0.7)
Thrombophlebitis	1 (0.7)
**Metabolic diseases**	19 (13.1)
Deficiency symptom*	14 (9.7)
Hyperlipidemia	4 (2.8)
Hyperuricemia	1 (0.7)
**Gastrointestinal diseases**	16 (11.0)
**Psychiatric diseases**	15 (10.4)
Depression	13 (9.0)
Anxiety	1 (0.7)
Schizoaffective disorder	1 (0.7)
**Bladder disorders**	7 (4.9)
**Osteoporosis**	4 (2.8)
**Eye diseases**	3 (2.1)
**Infections**	3 (2.1)
**Other**	8 (5.6)

N, number of patients.

* deficiency of Vitamin B12, D, iron, folic acid or calcium.

In the PwSI group, the average number of medications taken was approximately twice as high as in the Pw/oSI group (Pw/oSI vs. PwSI: 2.4 [SD 1.3] vs. 4.6 [SD 2.1]; Mann-Whitney U test: *p*<0.001). Similar results were found regarding the subsets of long-term medications (Pw/oSI vs. PwSI: 1.7 [SD 0.9] vs. 4.0 [SD 1.8]; Mann-Whitney U test: *p*<0.001) and prescription-only medications (Pw/oSI vs. PwSI: 1.6 [SD 0.9] vs. 3.5 [SD 1.6]; Mann-Whitney U test: *p*<0.001). No significant differences were found with respect to PRN medications, DMDs for MS or symptomatic drugs (*p*>0.05).

The data on route of drug administration showed that the average number of peroral medications taken was more than twice as high in PwSI than in Pw/oSI (Pw/oSI vs. PwSI: 1.5 [SD 1.4] vs. 3.6 [SD 2.1]; Mann-Whitney U test: *p*<0.001). This difference remained significant after FDR correction of the *p*-values. There were no further differences regarding the routes of administration.

## Discussion

The aim of this study was to examine the frequency of polypharmacy in RRMS patients in an outpatient setting and to determine possible influencing factors which foster polypharmacy. Studies on polypharmacy in MS patients are still rare [28–31]. Previous studies examined the issue with respect to fatigue and cognitive ability [31], quality of life and relapse rate [30] with regard to the use of antiepileptic drugs and antidepressants [28]. Our study, by contrast, included the whole range of medications and focused on polypharmacy-related factors, supplemented by a comparison of patients with and without secondary illnesses.

The average age of the study population was 43.0 years (SD 11.4), which is similar to that reported in other studies on polypharmacy in MS [29–31]. In the present study, we focused on patients with RRMS, as this represents the most frequent subtype of MS [37], for which a range of therapeutic options have been approved [4,11]. It is notable that, despite the young average age, almost 40% of the patients in our study were already retired. This can be explained by the limiting nature of the disease, for example through spasticity, fatigue, pain and sensory disturbances, which even at low EDSS scores can lead to incapacity for work.

As expected, the analysis of the clinical-neurological factors revealed a median EDSS score of 2.5, which lies within the lower range. This is due to the fact that we only considered RRMS cases, which are often treated in outpatient settings. Thus, the results of this study do not necessarily apply to other forms of MS. To generalize the findings, it will be essential to conduct further research with MS populations encompassing all subtypes of the disease.

There are numerous definitions of polypharmacy, including the classification into minor polypharmacy (two to four medications) and major polypharmacy (five or more medications) [38]. Moreover, polypharmacy is also used to denote the prescribing of two of more medications with the same therapeutic objective [39,40] or of two or more medications, which belong to the same chemical substance class [39]. Most commonly, it is defined as exceeding a previously determined number of medications [41]. The present study defined polypharmacy as the use of five or more medications, as this definition is often used in the literature [18–23]. While other studies did not distinguish between as-needed and long-term medications when defining polypharmacy, we took a more differentiated approach [28–31]. The analysis of polypharmacy according to long-term medications enabled us to focus on medications that are used permanently, but it neglected the PRN medications, which also play a substantial role in everyday life. As many people additionally take OTC medications [30], the analysis based on the whole range of medications may constitute a more comprehensive assessment.

In this study, polypharmacy was present in around one third (30.3%) of all patients when defined based on the full spectrum of medications. The second definition of polypharmacy, which took into account only long-term medications, served to place the focus on medications which are taken regularly and permanently, ignoring all others. According to this definition, the polypharmacy rate was a little lower (20.7%). These two polypharmacy rates fit in the middle of those found in other polypharmacy studies on MS. There, proportions of 14.9% [30], 32.9% [31], 38.3% [29] and 59% [28] have been reported, when defining polypharmacy as the use of five or more medications. The extreme rates of 14.9% and 59% resulted from disregarding immunotherapies [30] and from preselecting patients using antiepileptic drugs, respectively[28].

As expected, PwP were significantly older than Pw/oP (45.9 vs. 41.7 years) and twice as likely to be retired (59.1% vs. 30.7%). This might be attributable to the growing number of comorbidities and associated medical therapies with increasing age. Various studies have established that a higher age at MS diagnosis is associated with a higher likelihood of comorbidities [42,43]. Accordingly, the number of medications taken rises with age.

The novel observation that PwP had significantly more school years than Pw/oP (according to long-term medications only) suggests that lower performances in school are associated with an increased risk of polypharmacy. It has generally been shown that low school qualifications are generally associated with low health status and low health literacy [44]. Low health literacy can lead to incorrect medication use which provokes consequently a higher potential for non-adherence, that may limit drug efficacy, and side effects, that may lead to the prescription of new drugs. The social status (also dependent on education) plays a decisive role as well. Socially disadvantaged people are often smokers and physically less active and nutrition is less healthy than in people with higher social status and higher education [45]. All of these factors increase the risk of comorbidities, multi-drug exposure and therefore polypharmacy.

In answer to the question of which clinical factors might be associated with polypharmacy, we found the following: It was apparent that high EDSS scores were associated with polypharmacy (*p* = 0.029; OR = 1.440). Thus, for every 1.0 step on the EDSS, the risk of polypharmacy increased by 44.0%. Patients with higher EDSS scores are more likely to take symptomatic drugs (e.g. to maintain the ability to walk or to alleviate spasticity) and so the risk of polypharmacy is higher. Other MS studies on polypharmacy also found differences between Pw/oP and PwP regarding the degree of disability [30,31]. Moreover, the presence of secondary illnesses showed an association with polypharmacy in our data: The risk of polypharmacy was more than six times higher in PwSI than in Pw/oSI (*p*<0.001; OR = 6.293). Obviously, this is attributed to the fact that the occurrence of comorbidities generally means additional treatments and thus increases the risk of polypharmacy. The correlation analysis (Fig 4) also disclosed such dependencies in the data.

Subdividing the patients by the presence of comorbidities besides MS yielded approximately the same numbers of individuals per group: Pw/oSI (N = 68) and PwSI (N = 77). As expected, Pw/oSI were younger than PwSI, which reflects the rising frequency of comorbidities with age [42,43]. The risk of polypharmacy is strongly increased in PwSI, which implies a higher risk of drug interactions [24] and side effects. In this patient group, therefore, a well thought-out medication management is especially necessary.

Regarding the various medication categories, DMDs were, as expected, the most frequently used medications, followed by dietary supplements. Other studies also showed that taking dietary or herbal supplements is common in patients with MS [46–49]. Thus, supplements make up a large part of MS patients’ self-medication. Neglecting such prescription-free supplements by physicians carries a certain risk of possible drug interactions and side effects. The common use of analgesics against pain caused by MS or comorbidities was plausible too [15]. Furthermore, MS is associated with a higher osteoporosis risk [50] and female MS patients should be advised to use contraception during DMD treatment [51]. Thus, osteoporosis drugs and contraceptives are frequently used. In our data, dietary supplements, antihypertensives and gastrointestinal drugs were the most frequent medication groups which showed significant differences between Pw/oP and PwP. Patients with at least one cardiovascular condition (including hypertonia, coronary heart disease and heart failure) are likely to suffer from a further one [52]. This potentially increases the number of prescribed antihypertensives. Gastrointestinal drugs such as protein pump inhibitors are frequently prescribed particularly among older patients [53]. The most plausible reason for this may be the compensation of other therapeutic side effects in the gastrointestinal tract. These relationships may contribute to the observed associations of polypharmacy with the more frequent use of antihypertensives, gastrointestinal drugs and also dietary supplements.

Most patients (89.7%) examined in this study took drugs administered perorally. The average number of peroral medications taken differed significantly between Pw/oP and PwP (1.5 vs. 5.1; *p*<0.001). This may be explained by the fact that the majority of all drugs are administered in this way and it is also the most popular route of administration [54] as it is easy to understand, uncomplicated and well-manageable. In our study, the most prevalent comorbidities were thyroid diseases, hypertonia, gastrointestinal diseases and deficiency symptoms. Nearly all drugs taken against those comorbidities are administered perorally. Thus, the high number of medications taken by PwP, especially of peroral supplements, analgesics, osteoporosis drugs, thyroid drugs, antidepressants, antihypertensives and gastrointestinal drugs, is associated with a higher burden of comorbidity and disability, which again correlates with increased age. Furthermore, certain diseases require or permit combination therapies consisting of two or more drugs. For instance, hypertonia, glaucoma and asthma are often treated with combination therapies, which include peroral, conjunctival and inhalative medications.

For avoiding drug interactions and their clinical consequences, a well thought-out medication management that is based on the optimization of drug use is vital. A possibility for an adequate adaption or control lies in the analysis of the prescribed medications by the physician in order to check whether all medications are indeed essential and up-to-date for the respective patient. Moreover, evidence-based, non-drug approaches such as physiotherapy [55–57] and cognitive-behavioural talking therapy are also effective [58] and can serve to reduce medication complexities and to support treatment.

Some limitations of the present study should be mentioned. First, we focused only on MS patients with the relapsing-remitting subtype, meaning that the other subtypes were neglected. Second, the study was cross-sectional in design. Thus, it was not possible to make reliable statements about a longitudinal scenario. Third, glucocorticosteroids for acute relapse treatment were not included, as in our health care environment, treatments of relapses are generally conducted in the inpatient setting.

In conclusion, our study showed that polypharmacy plays an important role for MS patients and that it is associated with a higher EDSS score. RRMS patients with secondary illnesses are particularly affected by polypharmacy. Further evidence is needed on how polypharmacy poses an issue in the management of MS and this demands a prospective study of side effects, drug interactions and adherence problems.

## Supporting information

S1 Table Full dataset of relapsing-remitting MS patients (N = 145). DMD, disease-modifying drug; EDSS, expanded disability status scale; IT, immunotherapy; MS, multiple sclerosis; N, number of patients; OTC, over-the-counter; *PRN*, *pro re nata*.
(XLSX)
